# Inferring neutral biodiversity parameters using environmental DNA data sets

**DOI:** 10.1038/srep35644

**Published:** 2016-10-20

**Authors:** Guilhem Sommeria-Klein, Lucie Zinger, Pierre Taberlet, Eric Coissac, Jérôme Chave

**Affiliations:** 1 Université Toulouse 3 Paul Sabatier, CNRS, UMR 5174 Laboratoire Evolution et Diversité Biologique, F-31062 Toulouse, France; 2 Université Grenoble Alpes, CNRS, UMR 5553 Laboratoire d’Ecologie Alpine, F-38000 Grenoble, France

## Abstract

The DNA present in the environment is a unique and increasingly exploited source of information for conducting fast and standardized biodiversity assessments for any type of organisms. The datasets resulting from these surveys are however rarely compared to the quantitative predictions of biodiversity models. In this study, we simulate neutral taxa-abundance datasets, and artificially noise them by simulating noise terms typical of DNA-based biodiversity surveys. The resulting noised taxa abundances are used to assess whether the two parameters of Hubbell’s neutral theory of biodiversity can still be estimated. We find that parameters can be inferred provided that PCR noise on taxa abundances does not exceed a certain threshold. However, inference is seriously biased by the presence of artifactual taxa. The uneven contribution of organisms to environmental DNA owing to size differences and barcode copy number variability does not impede neutral parameter inference, provided that the number of sequence reads used for inference is smaller than the number of effectively sampled individuals. Hence, estimating neutral parameters from DNA-based taxa abundance patterns is possible but requires some caution. In studies that include empirical noise assessments, our comprehensive simulation benchmark provides objective criteria to evaluate the robustness of neutral parameter inference.

The observation of biodiversity patterns such as the diversity, relative abundance and spatial distribution of organisms underpins much of ecological theory^1^,^2^,^3^. Yet empirical measurements of these patterns are noisy. In all cases, some taxa are counted more effectively than others, and error is generated by misidentification. A major question is whether this noise is significant enough to undermine comparisons between empirical measurements and models^4^,^5^. This issue has recently taken on new significance following the advent of DNA-based biodiversity exploration methods, which are developing fast and hold the promise of rapid, repeatable and comprehensive biodiversity measurements^6^,^7^. Yet they are also less direct than classic biodiversity surveys and entail poorly assessed noise sources. In this study, we ask how the parameter estimates of Hubbell’s neutral theory, one of the most prominent quantitative biodiversity models of the last decade^3^,^8^,^9^, are affected by noise in taxa-abundance datasets. We focus on the type of noise generated in DNA-based surveys, and specifically in DNA metabarcoding surveys (see below)^6^, currently the most popular method for environmental DNA analysis. Nevertheless, our results can apply more generally.

DNA metabarcoding is a multi-taxa extension of the DNA-based identification of single specimen from tissue samples using a universal DNA-barcode sequence^10^. It consists in amplifying a short DNA barcode by PCR from the DNA extracted from an environmental sample (e.g. soil, water, bulk sample of organisms), and sequencing the product by high-throughput sequencing. This method is not restricted to the detection of known taxa and hence allows for comprehensive biodiversity measurement. DNA metabarcoding was initially developed to study bacterial communities^11^,^12^,^13^,^14^, but has since been extended to many other groups including archaea^15^ and eukaryotic clades (e.g. plants, earthworms, insects, fungi^16^,^17^,^18^,^19^). It is hence now possible to study patterns of diversity across all domains of life^20^,^21^. However, DNA metabarcoding observations have seldom been compared to the predictions of biodiversity models^3^,^22^.

Over the past decade, the neutral theory of biodiversity has represented a significant advance in interpreting empirical biodiversity patterns within an ecological guild^3^,^8^,^9^. Hubbell’s neutral model is simple, easily generates biodiversity patterns, allows for exact maximum-likelihood parameter inference from taxa-abundance distributions, and neutral predictions on taxa-abundance distributions compare well with empirical surveys^23^,^24^,^25^. In Hubbell’s model, sites vacated by the death of an individual are replaced by the offspring of local individuals or by immigrants. Birth, death and immigration all occur irrespective of the taxon the organism belongs to (neutrality hypothesis). Immigrants are drawn from a much larger (regional) pool of individuals, and the addition of new taxa in the regional pool is made possible by (rare) speciation events. Hubbell’s model has two parameters: *θ* describes the taxon diversity of the regional pool, and *m* is the immigration rate from the regional pool into the sampled community (see Supplementary Note 1).

The predictions of Hubbell’s neutral model have so far been primarily compared to integrative patterns obtained for macroorganisms using classic census data, such as the abundance distribution of tropical forest trees^3^. Some studies have also applied neutral models to environmental DNA data to interpret the composition of microbial communities. Sloan *et al*.^26^,^27^ and Woodcock *et al*.^28^ developed a continuous approximation to Hubbell’s model adapted to large-sized bacterial populations. They focused on estimating the rate of immigration into the local community independently of assumptions on the regional pool of taxa, by comparing taxa occurrence in multiple samples^26^,^29^,^30^,^31^,^32^ or by measuring the turnover of taxa over time^33^. The composition of many microbial communities was found to be compatible with stochastic immigration of taxa of equivalent fitness from a regional pool, at odds with the classic assumption that deterministic niche sorting explains the assemblage of microbial communities^34^,^35^. Another approach is to simultaneously estimate the diversity and immigration parameters by fitting the taxa-abundance distribution, as it has been commonly done for classic censuses of macroorganisms. Dumbrell *et al*.^36^ and Lee *et al*.^37^ did so on fungal and bacterial DNA data using maximum-likelihood parameter inference based on the exact Etienne sampling formulas^23^,^38^,^39^, while Harris *et al*.^40^ followed an approximate Bayesian approach inspired by the field of machine learning.

Most DNA-based studies comparing empirical abundance patterns to the predictions of neutral models have been limited by the poor detectability of rare taxa owing to the methods used (Sanger sequencing, DGGE, t-RFLP, ARISA). High-throughput sequencing now allows for improved sampling and provides better quality data. Nevertheless, metabarcoding data are not directly comparable with classic census data owing to both experimental and biological factors. First, both PCR amplification and sequencing produce artifacts. During the PCR amplification, DNA polymerase makes mistakes when replicating DNA strands, at a rate that depends on enzyme types. DNA strands suffer further damage during the high-temperature denaturation step^41^,^42^,^43^. Furthermore, Illumina sequencing generates between 10^−3^ and 10^−2^ errors per base pair^44^. Clustering algorithms are used to cluster the reads displaying errors with respect to the original sequence into a single Molecular Operational Taxonomic Unit (MOTU)^45^,^46^,^47^. While these approaches strongly reduce the number of artifacts in the data, they do not exclude artifactual MOTUs that are more difficult to detect (e.g. chimerical fragments, highly degraded sequences). Second, unbalanced PCR amplification and sequencing among taxa distorts the relative abundances of MOTUs^48^,^49^,^50^,^51^. Third, relative abundances are further biased by noise sources inherent to the use of DNA barcodes, such as the strong variability of the barcode copy number among taxa^52^,^53^. This problem is even more serious for multicellular organisms because the read count should also depend on cell abundance. Abundances are further biased by the variable rate of DNA release into the environment through excreted, sloughed or decaying material^54^,^55^,^56^.

In this paper, we conduct simulations to address how the sources of uncertainty mentioned above may distort parameter estimates in Hubbell’s neutral theory, and we discuss the conceptual differences between individual-based and environmental DNA approaches to the measurement of biodiversity. We ask the following questions: (1) what is the effect of artifactual MOTUs and abundance noise on estimating the neutral diversity parameter? (2) Can we use the same approach for multicellular as for unicellular organisms? (3) What are the effects of the different noise sources on neutral parameter inference when accounting for dispersal limitation?

## Methods

### Sampling from Hubbell’s neutral model

We generated samples of *J* individuals following the stationary taxa-abundance distribution of Hubbell’s neutral model. The immigration from the regional pool of diversity parameter *θ* into the sampled community can be either characterized by the immigration rate *m* or by the normalized immigration parameter 
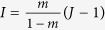
 that does not depend on the sample size *J* and is thus invariant by sampling. If *m* ≪ 1, *I* is approximated by the product *Jm*, noted *N*_*T*_*m* in Sloan *et al*.^26^,^27^.

We first assumed no dispersal limitation (i.e. *m* = 1). We generated a sample by running *J* times the following algorithm parameterized by *θ*: at step *j*, draw individual *j* + *1* from a new taxon with probability *θ*/(*j* + *θ*), or draw one of the *j* individuals already present and add an individual *j* + *1* of the same taxon. This algorithm, due to Hoppe^57^, partitions *J* individuals into a random number *T* of taxa according to the Ewens distribution of parameter *θ* 58.

We then generated samples from a dispersal-limited neutral community using the two-step procedure provided in Etienne^23^ which partitions *J* individuals into a random number *T* of taxa. First, we run *J* times Hoppe’s algorithm as described above but with parameter *I*, so as to partition the *J* individuals into *A* immigrating ancestors. Second, we run *A* times the algorithm with parameter *θ*, so as to partition the *A* immigrating ancestor into *T* taxa, thus taking into account the taxa-abundance distribution in the regional pool. Finally, we assign the *J* individuals to the taxonomic identity of their immigrating ancestor.

We generated samples of *J* = 10^5^ individuals. We explored a realistic range of parameter values: *θ* in [1, 500] and *m* in [0.001, 1].

### Simulating noise in DNA sequence reads: experimental noise

We simulated the DNA metabarcoding procedure by sampling *N* sequence reads from the relative taxa abundances of the neutral model, possibly after modifying the relative abundances according to simulated noise sources (see below). We present the results obtained for the value *N* = 10^4^, a typical number of Illumina sequence reads for one environmental sample.

In order to test the effect of misidentification bias on neutral parameter inference, we added artifactual MOTUs to the data, while keeping the number of reads constant. We assumed that each true MOTU with a read abundance *r* generates a random number of artifactual MOTUs, drawn from a multinomial distribution with weight *r*. We added either singletons, or MOTUs with larger read abundances. We obtained an example of artifactual MOTUs with realistic abundance structure from a benchmark experiment (see below and Supplementary Methods). Drawing on these empirical data, we simulated read abundances in the following way: each artifactual MOTU was assumed to have an abundance of 1 read if *r* < 50, or an abundance *x* if *r* ≥ 50, where *x* lies between 1 and *r*/50 with a probability density 
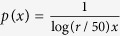
.

Molecular experimental procedures introduce biases also in read abundances, because the efficiency of PCR amplification and sequencing is variable across MOTUs. For instance, PCR amplification is less efficient if PCR priming sites differ from the primer sequence^48^, or if the barcode sequence is too long or GC-rich^50^. As a result, the read abundance distribution of MOTUs is noised with respect to the DNA barcode abundance distribution in the sample. We assumed that the noise takes the form of a lognormally distributed multiplicative noise on relative abundances, with mean 1 and log standard deviation *σ*_*log*_. This choice is parsimonious because this noise is predominantly due to PCR^50^, and the multiplicative amplification of DNA strands by PCR generates a multiplicative noise on abundances. This multiplicative noise can be further assumed to result from the product of random independent variables and thus to be lognormally distributed by virtue of the central limit theorem. We tested the effect of noise intensity *σ*_*log*_ on neutral parameter inference. For completeness, we also tested the effect of an additive Gaussian noise of standard deviation *σ*_*add*_ on MOTUs relative abundances, for different *σ*_*add*_ values. This type of noise can be regarded as simulating the noise generated in the sequencing step.

To illustrate our modelling choices with empirical data, we produced a benchmark dataset obtained by mixing the DNA of 16 plant species in known quantities. The experiment and its results are detailed in the Supplementary Methods. After following standard data curation protocols, we found that the dataset contained 33% of artifactual MOTUs and displayed a lognormally distributed multiplicative noise on relative abundances of log standard deviation *σ*_*log*_ = 1.2. We reported these values on the figures as examples of realistic noise intensities.

### Simulating noise in DNA sequence reads: ‘biological’ noise

Irrespective of experimental noise, variability in the number of barcode copies per individual may cause bias in the interpretation of read abundances. For bacteria (16S rDNA) or protists (18S rDNA), barcode copy number variability in nuclear DNA is an important contribution to abundance noise^52^,^53^: Kembel *et al*.^52^ found that the barcode copy number of the 16S rDNA gene follows a zero-truncated Poisson distribution of parameter *λ* = 4 across a range of bacterial clades. For multicellular eukaryotes, organellic barcodes are typically used, and they similarly display variable copy numbers per cell across taxa and tissue types. To assess this issue, we tested how a zero-truncated Poisson-distributed multiplicative noise affects neutral parameter inference, for various values of the parameter *λ*. The intensity of this noise is measured by the coefficient of variation (i.e., standard deviation over mean) of the zero-truncated Poisson distribution. Since it reaches a maximum at *λ* = 1.8, noise intensity is maximal for this value.

For multicellular organisms, the variability in the number of barcode copies per individual is further amplified because the number of cells may vary vastly across individuals, owing to body-size differences. We simulated size differences between individuals following a simple and generic approach. As in O’Dwyer *et al*.^59^, we assumed that all individuals, irrespective of the taxon they belong to, grow in size over time at a constant rate *g* from an initial number of cells *n*_*0*_ at birth, and die at a constant rate *d*. The stationary probability density *p*_*ind*_(*n*) of having a number *n* of cells for a randomly chosen individual is given by the solution of the von Foerster equation^59^: 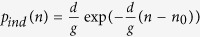 (see Supplementary Note 2). We used this distribution to draw a number *n* of cells between *n*_*0*_ and infinity for each individual, and modified the MOTUs relative abundances accordingly. Note that we simulated size differences between individuals and not between taxa, which would have been akin to simulating a multiplicative noise on taxa abundances as above. We tested the effect on neutral parameter inference for a range of values of 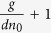, the ratio of the mean cell number 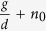 divided by the initial cell number *n*_*0*_. Noise intensity is measured by the coefficient of variation 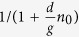 of the probability density *p*_*ind*_(*n*). It is bounded by 1 for 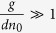, which corresponds to the case of taxa spanning large ranges of body sizes, such as trees or vertebrates.

Organisms may be entirely contained in the environmental sample if they are sufficiently small, or when DNA is extracted from a mixture of directly sampled live organisms, such as insects from a light trap (bulk samples)^18^. However, in most cases, only small fractions of these organisms are sampled (e.g. roots, pollen, seeds, spores, faeces, and different secretion types), or even only extracellular DNA resulting from cell death and subsequent destruction of cell structure^6^,^60^. Thus, the abundance distribution of environmental DNA also depends on the kinetics of DNA release and degradation in the environment. We assumed that this dynamics is fast with respect to changes in community composition, so that the ‘stock’ of environmental DNA is in a steady state. Under this assumption, the rate of DNA release through the death of organisms is roughly proportional to the total number of cells of the currently living individuals. In addition, the rate of environmental DNA release by a living organism reflects its metabolic rate and we assumed it to scale as the power 3/4 of body mass (or cell number), as predicted by the metabolic theory of ecology^61^. DNA degradation rate was assumed uniform across individuals. Even though we focus here on multicellular organisms, unicellular organisms do excrete DNA material and differ in metabolic rates as well.

Based on the assumptions of the previous paragraph, we simulated the abundance distribution of environmental DNA as follows. We (1) generated a neutral sample of individuals, (2) assigned a number of cells *n* between *n*_*0*_ and infinity to each individual as above, (3) counted a first contribution *dn* of each individual to the stock of environmental DNA, with *d* the death rate, (4) and counted a second contribution *r*_0_*n*^3/4^ of each individual to the stock of environmental DNA, with *r*_*0*_ the rate of DNA release for a hypothetical one-cell individual. Thus, environmental DNA abundance per individual is proportional to 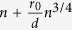 rather than *n*. We tested the effect on neutral parameter inference by varying 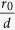, the parameter controlling the relative contribution of living and dead organisms to environmental DNA.

### Estimating the neutral model parameters from the taxa-abundance distribution

We estimated the parameters of Hubbell’s neutral model by maximum-likelihood inference from the simulated taxa-abundance distribution for a number of simulated noise sources. To test the influence of noise, we compared the estimated parameter values 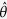 and 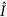 with the values of *θ* and *I* used to generate the initial samples of individuals. For each set of parameters and noise intensity, we generated 100 simulated samples. We reported the mean and standard deviation of the relative biases 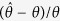 and 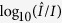 over the 100 realizations.

In the absence of dispersal limitation, the Ewens distribution permits the inference of *θ* by likelihood maximization. The maximum-likelihood estimator of *θ*, hereafter referred to as the Ewens estimator, is implicitly given by 
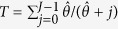
 as a function of the number *T* of taxa and the number *J* of individuals^58^. In the dispersal-limited case, the Etienne distribution provides an exact likelihood expression for the simultaneous inference of *θ* and *I*^23^, as implemented in the software Tetame^62^. As noted previously in the literature, the likelihood landscape of the Etienne formula often displays two local maxima^25^,^63^. To find the true parameter values, we first estimated *θ* using the Ewens estimator, and selected the local maximum with the *θ* estimate closest to the value yielded by the Ewens estimator. Prior to these analyses, we tested the performances of both estimators on unbiased neutral data depending on parameter values and sample size (see Supplementary Note 3).

In typical environmental DNA data, the number *J* of individuals in the sample is unknown. As already done in previous studies^37^, we used the number of sequence reads as an effective number of individuals. This is possible owing to a mathematical property of the Ewens and Etienne distributions: both distributions are invariant by sampling without replacement^24^, hence maximum-likelihood inference yields the same results on any random sample from the community, and on any random subsample from an initial sample (up to a possible bias in the estimator). As a consequence, read abundances can be used for neutral parameter inference, as long as the reads can be regarded as forming a subsample without replacement of the initial individuals. This assumption is however not always verified in empirical data (see Discussion). The invariance property of Etienne distribution only holds if the distribution is expressed as a function of *I*, therefore we used here the immigration parameter *I* instead of *m* for the purpose of inference. In the following, *m* always refers to the value in the initial sample of *J* individuals.

In the absence of dispersal limitation, *θ* can also be estimated from the slope of the ranked log-abundance curve, a method that has the advantage of being independent of *J*. Indeed, the logarithm of E[*P*_*i*_], the expected relative abundance of the *i*^th^ most abundant taxon, is given by^64^: log(E[*P*_*i*_]) = −log*θ* − *i*log(1 + 1/*θ*). For simulated abundance noise, we estimated *θ* using this method in addition to Ewens estimator. We restricted the linear regression to the linear domain of the ranked log-abundance curve. We also compared the performance of both inference methods in the absence of simulated noise for samples of 10^2^, 10^3^, 10^4^ and 10^5^ sequence reads and for initial samples of individuals of different sizes (see Supplementary Note 4).

## Results

**Figure 1:**
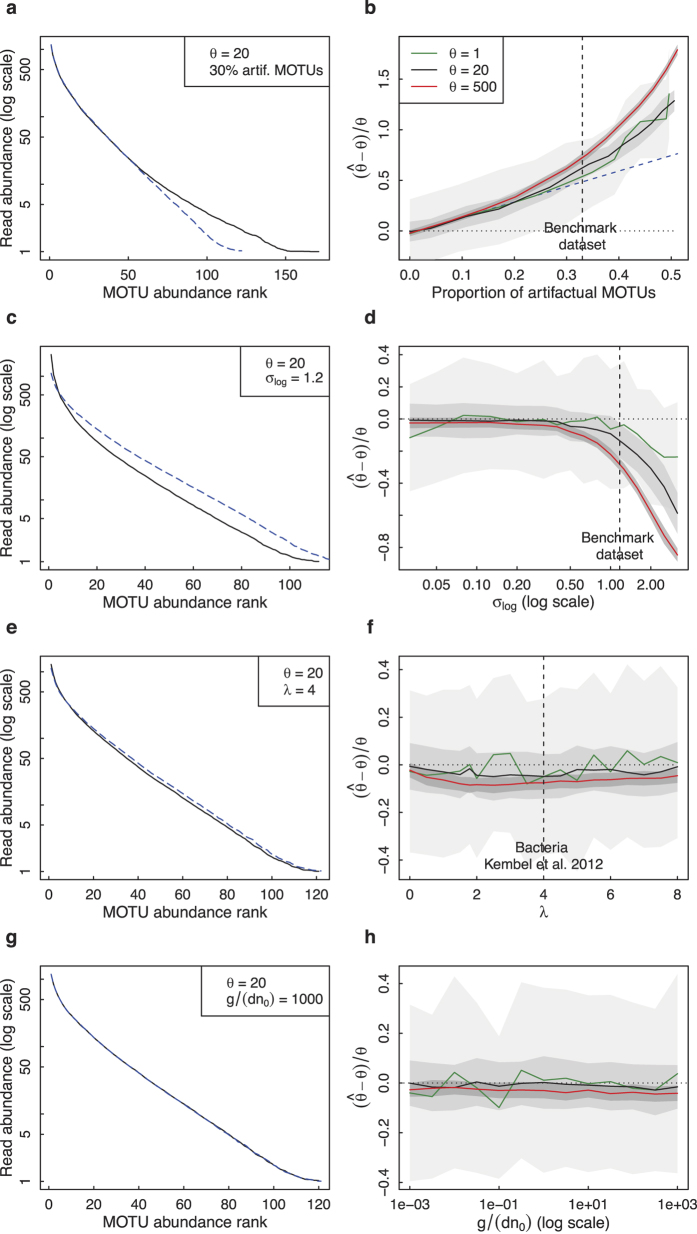
Neutral parameter inference without dispersal limitation. Left panels: mean MOTU rank- abundance distributions over 100 realizations for *θ* = 20 in a 10^4^-read sample, without (dashed blue line) and with (black line) simulated noise: (**a**) 30% artifactual MOTUs added (as measured in benchmark dataset), (**c**) multiplicative lognormal noise of log standard deviation *σ*_*log*_ = 1.2 (as measured in benchmark dataset), (**e**) multiplicative zero-truncated Poisson noise simulating barcode copy number variability (Poisson parameter *λ* = 4; cf. Kembel *et al*.^52^), and (**g**) size structure among individuals, for a ratio 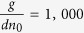 (mean body mass over birth mass). Right panels: mean and standard deviation over 100 realizations of the relative bias on the *θ* estimate in a 10^4^-read sample, for *θ* = 1 (green), *θ* = 20 (black) and *θ* = 500 (red), as a function of (**b**) the proportion of artifactual MOTUs (dashed blue line underlines the linear dependence), (**d**) the lognormal noise intensity *σ*_*log*_, (**f**) the Poisson parameter *λ*, and (**h**) the ratio 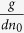.

We first included artifactual MOTUs in a simulated sample and tested the effect on estimating the diversity parameter *θ* of the neutral model without dispersal limitation. The relative bias 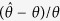 increased with the proportion of artifactual MOTUs, first linearly and then faster than linearly (Fig. 1a,b). It did not depend on the initial *θ* value or on the read abundance of the introduced artifactual MOTUs. The standard deviation of 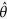 was not modified by the presence of artifactual MOTUs.

Next, we simulated PCR noise, modelled as a lognormally distributed multiplicative noise with log standard deviation *σ*_*log*_. This noise had no effect on the inference of the *θ* parameter below a threshold *σ*_*log*,*th*_. For *σ*_*log*_ > *σ*_*log*,*th*_, *θ* was underestimated. The value of *σ*_*log*,*th*_ decreased with increasing *θ* but remained of the order of 1 for *θ* between 1 and 500 (*σ*_*log*,*th*_ ≈ 5 for *θ* = 1 and *σ*_*log*,*th*_ ≈ 0.5 for *θ* = 500; see Fig. 1c,d). We also applied an additive Gaussian noise of standard deviation *σ*_*add*_ to the relative abundances. This type of noise introduced a bias in 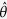 for values of *σ*_*add*_ at least one order of magnitude larger than the relative abundance of the least abundant MOTUs (Supplementary Fig. S1). Neither type of noise affected the standard deviation of 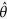 (Fig. 1, Supplementary Fig. S1). These results held both in maximum-likelihood inference and when using linear regression on the ranked log-abundance.

We then simulated the variability in barcode copy number by applying a multiplicative noise distributed according to a zero-truncated Poisson distribution. This type of noise had no effect on *θ* inference, even for the maximum noise intensity at *λ* = 1.8 (Fig. 1e,f). We accounted for body size differences by assuming a steadily growing cell number *n* over the course of an individual’s life, and by varying the ratio 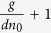 of the mean number of cells 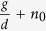 divided by the initial number of cells *n*_*0*_. We found that this ratio had no effect on the mean and standard deviation of 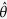, even at large values (Fig. 1g,h). We also tested the effect of assigning an environmental DNA mass proportional to 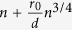 to individuals (where *n* is the cell number) to reflect the joint effect of mortality (*n* term) and cellular turnover (*n*^3/4^ term, proportional to metabolic rate). We did not find any effect on *θ* inference even for large values of 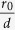 (Supplementary Fig. S1).

**Figure 2:**
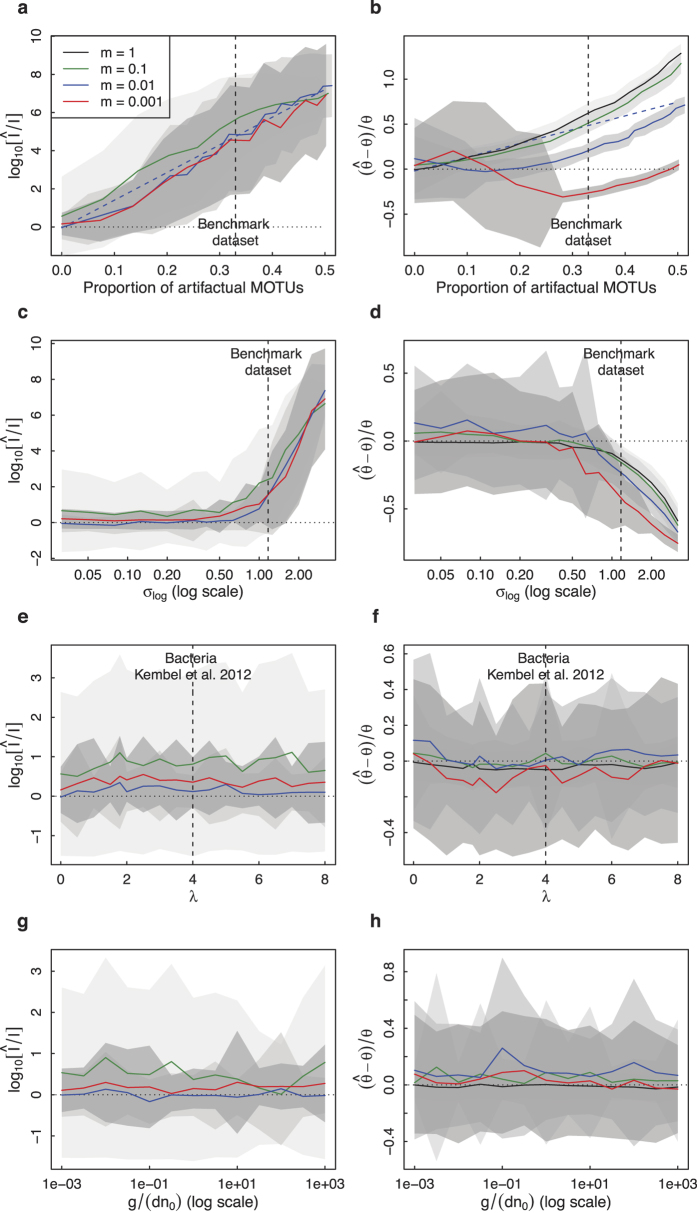
Neutral parameter inference in the presence of dispersal limitation. We simulated a 10^4^-read sample and computed the mean and standard deviation over 100 realizations of 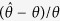 and 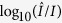. Results are plotted for *θ* = 20 and for *m* = 1 (black), *m* = 0.1 (green), *m* = 0.01 (blue) and *m* = 0.001 (red). Panels a,b: variation with the proportion of artifactual MOTUs (dashed blue line underlines the linear dependence). Panels c,d: variation with the log standard deviation *σ*_*log*_ of a multiplicative lognormal noise on relative abundances. Panels e,f: variation with the parameter *λ* of a multiplicative zero-truncated Poisson noise. Panels g,h: variation with body size ratio 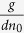.

Finally, we replicated the analysis in the presence of dispersal limitation (i.e. assuming that *m* < 1). We found that the dispersal-limited maximum-likelihood estimator can be strongly biased even in the absence of simulated noise when dispersal limitation is too strong or too weak, especially for large *θ* values (see Supplementary Note 3). Therefore, we limited ourselves to parameter values that could be well estimated in the absence of simulated noise. Provided that the immigration rate was large enough (*m* > 0.1), the relative bias 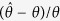 depended on the proportion of artifactual MOTUs similarly to the *m* = 1 case. For lower values of *m*, the dependence of 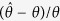 on the proportion of artifactual MOTUs was even stronger (Fig. 2a,b). The relative bias 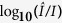 on the normalized immigration parameter increased linearly with the proportion of artifactual MOTUs. Applying a lognormal multiplicative noise of log standard deviation *σ*_*log*_ on MOTUs relative abundances did not bias the estimation of (*θ, I*) below a noise threshold *σ*_*log*,*th*_ identical to the one found without dispersal limitation. The threshold *σ*_*log*,*th*_ decreased only slightly with decreasing *m* value. Above *σ*_*log*,*th*_, *θ* was underestimated and *I* overestimated (Fig. 2c,d). Applying an additive Gaussian noise of standard deviation *σ*_*add*_ to the relative abundances biased the parameter estimates when *σ*_*add*_ was larger than the relative abundance of the least abundant MOTUs (Supplementary Fig. S2). A multiplicative noise distributed according to a zero-truncated Poisson had no influence on the parameter estimates (Fig. 2e,f), and likewise an exponentially distributed number of cells still had no effect on parameter inference in the dispersal-limited case (Fig. 2g,h, Supplementary Fig. S2).

## Discussion

Although they provide an unparalleled amount of information, biodiversity studies based on environmental DNA also have limitations. One of them is that the abundance of sequence reads corresponding to a given molecular taxonomic unit does not necessarily reflect the true population abundance of the corresponding taxon. Our analysis offers a quantitative assessment of the importance of this issue in attempting to relate environmental DNA datasets with theoretical model predictions.

Our goal was to assess when amplicon-based DNA read abundance data can offer biological insights into Hubbell’s neutral theory’s predictions. We selected Hubbell’s model over other models predicting taxa-abundance distributions because it incorporates a number of key features for any biodiversity model such as demographic stochasticity and dispersal limitation^65^. Estimating the parameters *θ* and *m* of the neutral model is useful in interpreting biodiversity patterns even if the community is not governed by purely neutral mechanisms^62^. Indeed, *θ* is closely related to Fisher’s biodiversity index, and is an unbiased index of biodiversity, while *m* quantifies how the local sample is connected to its surroundings. We simulated taxa abundance datasets from a neutral model and ‘noised’ them using a range of plausible noise types and intensities. We showed that the parameters *θ* and *I* could still be reliably estimated by maximum likelihood inference from the simulated sequence reads, provided that artifactual MOTUs are rare, and that lognormal noise on relative read abundances is below a log standard deviation threshold that depends on *θ*. We also showed that under our modelling assumptions, neutral inference is unbiased for assemblages of multicellular organisms and for variable barcode copy numbers. Finally we found that the noise terms had a similar effect on parameter inference when fitting the one-parameter version of the model (without dispersal limitation) and when fitting Hubbell’s dispersal-limited model.

One of the major differences between environmental DNA surveys and classic biodiversity surveys is that the number of sampled individuals is usually not measured. Yet, most biodiversity measures assume the knowledge of the organisms’ sample size. To solve this problem, we assumed in our simulations that the number of reads is several times smaller than the number of effectively sampled individuals: *N* = 10^4^ sequence reads for *J* = 10^5^ initial individuals. Under this assumption, sequence reads may be seen as a random subsample of the individuals, and because the maximum-likelihood approach of the neutral theory relies on sampling formulas that are invariant under subsampling, it follows that the inference on reads is unbiased (see Supplementary Note 4). Generating a larger number of individuals did not alter our results but was computationally prohibitive with our algorithm.

The assumption that the number of sampled individuals exceeds that of sequence reads is reasonable for prokaryotes^66^ and microorganisms in general, but is unrealistic for larger organisms. One empirical method to test whether the sequencing data meet the requirement for neutral maximum-likelihood inference is to take a smaller subsample of reads and check that the parameter estimates are unchanged. If not, one should decrease sample size until stability is achieved (see Supplementary Note 4). If environmental DNA data do not consist in a discrete number of reads, as is the case in t-RFLP and ARISA, an arbitrarily set sample size may be used^37^. The number of individuals can also be estimated empirically, as in Woodcock *et al*.^28^ or Dumbrell *et al*.^36^. In the neutral model without dispersal limitation, a more straightforward approach is to infer *θ* from the slope of the ranked log-abundance distribution, but this requires an arbitrary delimitation of the linear domain of the curve, and it is reliable only if the read sample is large enough and contains a large enough taxonomic diversity. A general rule is that the sampling scheme should be suited to the size and spatial density of the target organisms: for large organisms, multiple spatially distributed environmental samples should be pooled so as to sample a sufficiently large number of individuals. For instance, capturing the abundance distribution of plant taxa from soil DNA samples requires pooling a sufficient number of soil samples over a sufficiently large area.

When accounting for dispersal limitation, a single sample of sequence reads does not always provide enough information to reliably infer both *θ* and *I* from the taxa-abundance distribution, even in the absence of additional noise source. The maximum-likelihood estimator may be strongly biased when the immigration rate into the local community is either too low or too high, and increasingly so for larger *θ* □□□□□□ (see Supplementary Note 3). Since these biases decrease with larger read sample size, the number of sequence reads should be as large as possible, as long as it does not preclude using the sequence reads for parameter inference (see previous paragraph). Moreover, in order to avoid bias in the case of weak dispersal limitation, the Ewens estimator should be favoured whenever it yields a higher likelihood value than the dispersal-limited estimator.

In practice, environmental DNA studies often sample the same regional species pool in different locations, which allows for more robust multi-sample maximum-likelihood inference^38^,^39^. It should be noted however that exact maximum-likelihood inference can be computationally prohibitive in the dispersal-limited case for larger numbers of reads than we used in this study or in the case of a multi-sample approach with large read samples^37^. Continuous approximations drawing on the work of Sloan *et al*.^26^,^27^ and Woodcock *et al*.^28^ might then be preferred, such as the Bayesian formulation of Harris *et al*.^40^.

Our analysis reveals that the presence of artifactual MOTUs is the most detrimental to neutral parameter inference. Bioinformatics methods aiming at limiting the number of artifactual MOTUs should be carefully applied to the sequencing data before any attempt at estimating biodiversity indices^45^,^46^,^47^. However, these methods do not guarantee a complete filtering of artifactual MOTUs from empirical datasets. In particular, chimeric sequences formed at the PCR stage may be misconstrued as MOTUs. Because these sequences are generated by rare PCR replication errors, they should be predominantly represented by few reads. Thus one strategy for removing artifactual MOTUs consists in ignoring all MOTUs below an empirically set abundance threshold. However, in doing so, we lose the information on the relationship between the number of reads and the number of MOTUs. Hence we suggest that a more satisfactory method to mitigate this problem is to take a sufficiently small subsample of the sequence reads so as to trim out the artifactual MOTUs.

The presence of artifactual MOTUs in our simulated taxa assemblages manifests itself by a break in the slope of the ranked log-abundance curve (Fig. 1a, see also Fig. S3 in Supplementary Methods). Thus, the adequate subsample size for an empirical dataset may be chosen so as to trim out the MOTUs with abundances below an observed break in the ranked log-abundance curve. Another finding of our study is that for the same proportion of artifactual MOTUs, the *θ* estimate has a similar relative bias across *θ* values and the *I* estimate a similar relative bias across *I* values. Therefore, if artifactual MOTUs cannot be entirely excluded in an environmental DNA dataset, conclusions should be based on ratios of neutral parameter estimates among samples rather than on absolute values.

We modelled PCR noise using a lognormally distributed multiplicative noise term. We found a threshold noise value beyond which the inference of the neutral parameters becomes biased. This threshold was found to be lower for larger *θ* values. For instance, the empirical noise intensity σ_*log*_ = 1.2 measured on our benchmark dataset was near or below the threshold *σ*_*log*,*th*_ for *θ* values up to ca. *θ* = 20, while for larger *θ* values, it was responsible for a moderate underestimation of *θ* (20% for *θ* = 500) and for a serious overestimation of *I.* Nevertheless, our benchmark dataset was here used for illustrative purposes, and noise intensity may differ in other datasets. In metabarcoding studies, noise intensity likely depends on the barcode, taxonomic group and wet laboratory protocol. Therefore we strongly advise to include at least one benchmark dataset as part of any environmental DNA study to quantify noise intensity. Empirical noise assessments can then be compared to our simulation results.

We also simulated a Gaussian additive noise on abundance data and found that it had a disproportionate effect on the least abundant MOTUs, thus distorting the taxa-abundance distribution: parameter inference was biased if the standard deviation of the noise was larger than the abundance of the least abundant MOTUs. Here again, it is possible to correct for this type of noise in empirical datasets by subsampling the sequence reads. Additive noise can be considered to model the abundance noise generated by the sequencing step or by a single PCR cycle, while the succession of several PCR cycles produces a multiplicative abundance noise.

Another potential bias is due to the indirect relationship between the number of DNA barcode sequences in the sample and the number of sampled individuals. In particular, in the case of multicellular individuals, some of them may contribute disproportionately more than others. Given the variability and complexity of the associated noise structure, we chose to follow a modelling approach retaining as much generality as possible. We size-biased our samples by assuming that DNA availability in the environment is proportional to body mass, or to the turnover of body mass (i.e. the metabolic rate). We found that neutral parameter estimates are not modified by size structure in the community, irrespective of how strongly structured the community is, which is an interesting and general result.

Our approach to accounting for body size is directly inspired from the size-structured neutral model of O’Dwyer *et al*.^59^. This model integrates the growth of individuals into a neutral population dynamics without dispersal limitation, and may offer analytical predictions for the neutral “Species Biomass Distribution” (SBD) while accounting for the dependence of birth, death and growth rates on the size of individuals. When individuals grow in body size at a constant rate and neither birth nor death rates depend on size, this model predicts the same SBD as obtained analytically under our assumption of independent exponentially distributed sizes (see Supplementary Note 2). Our choice of a rate of environmental DNA release scaling with the 3/4^th^ power of body mass is motivated by a prediction of the metabolic theory of ecology, which relates metabolic rate to body mass in one of the few general laws of ecology^61^.

Even though our modelling approach derives from theoretical considerations, it is also supported by some empirical evidence: it has been shown that the rate of DNA detection in the environment is biased by the size of organisms^54^,^55^,^56^, and the fact that DNA abundance should scale non-linearly with body mass has been experimentally verified in fishes^55^. Nevertheless, the noise introduced by size structure, fragments of organisms and extracellular DNA certainly has a far more complicated structure than we simulated. For instance, rates of DNA release into the environment and of DNA degradation both depend on taxa and on local conditions, and fluctuate temporally^60^,^67^,^68^. Moreover, the uneven spatial distribution of environmental DNA may prevent properly sampling the taxa-abundance distribution in the community, especially if whole pieces of living or decaying multicellular organisms are contained in the environmental sample. Pooling multiple spatially distributed samples should help average out local heterogeneity.

In this study, we considered that departure of the number of DNA barcode reads from the real taxon abundance is sources of bias. However, these sources of bias may be generally seen as the accumulation of mutations during replication. In ecology, the only type of replication taken into consideration is demography, but DNA metabarcoding data are also the result of cellular and PCR replication processes. Since the assumptions of the neutral theory are generic and apply to any collection of replicating, mutating, and potentially dispersing entities, we could replace individual organisms by DNA barcodes as our basic replicating entities, and reinterpret the neutral parameters accordingly. As a consequence, we expect the taxa-abundance structure predicted by neutral theory to be robust as long as the DNA barcodes do not differ too much in their replicating, mutating and dispersing abilities.

This study demonstrates that inferring the parameters of Hubbell’s neutral model from the taxa-abundance distribution is possible even in noised biodiversity datasets. We tested this hypothesis for a range of biologically plausible noise terms on simulated metabarcoding data, and we provide guidance for neutral parameter inference from such data. Our results indicate that whether an environmental DNA dataset really reflects the sampled community depends on noise intensity. They also suggest that this question can be answered by computing simple metrics on a benchmark dataset and comparing them to our simulations. The only way to quantify the noise level is to conduct careful benchmarking experiments, which will depend on the exact sampling and analysis protocol.

## Additional Information

**How to cite this article**: Sommeria-Klein, G. *et al*. Inferring neutral biodiversity parameters using environmental DNA data sets. *Sci. Rep.* **6**, 35644; doi: 10.1038/srep35644 (2016).

## Supplementary Material

Supplementary Information
