# Supplementary material for: Inferring neutral biodiversity parameters using environmental DNA data sets
Source: Sci Rep. 2016 Oct 20;6:35644. doi: 10.1038/srep35644 (PMC5071827; doi:10.1038/srep35644)
Supplement: Supplementary Information [file srep35644-s1.pdf]

Inferring neutral biodiversity parameters using environmental DNA data sets - Supplementary Information

Authors: Guilhem Sommeria-Klein[1,*], Lucie Zinger[1], Pierre Taberlet[2], Eric Coissac[2], Jérôme Chave[1]

[1] *Université Toulouse 3 Paul Sabatier, CNRS, UMR 5174 Laboratoire Evolution et Diversité Biologique, F-31062 Toulouse, France.*
[2] *Université Grenoble Alpes, CNRS, UMR 5553 Laboratoire d'Ecologie Alpine, F-38000 Grenoble, France.*
* Corresponding author:
Laboratoire Evolution et Diversité Biologique UMR CNRS-UPS 5174, 118 route de Narbonne, 31062 Toulouse cedex 9, France
E-mail address: guilhem@sommeria-klein.fr

Supplementary Figure 1

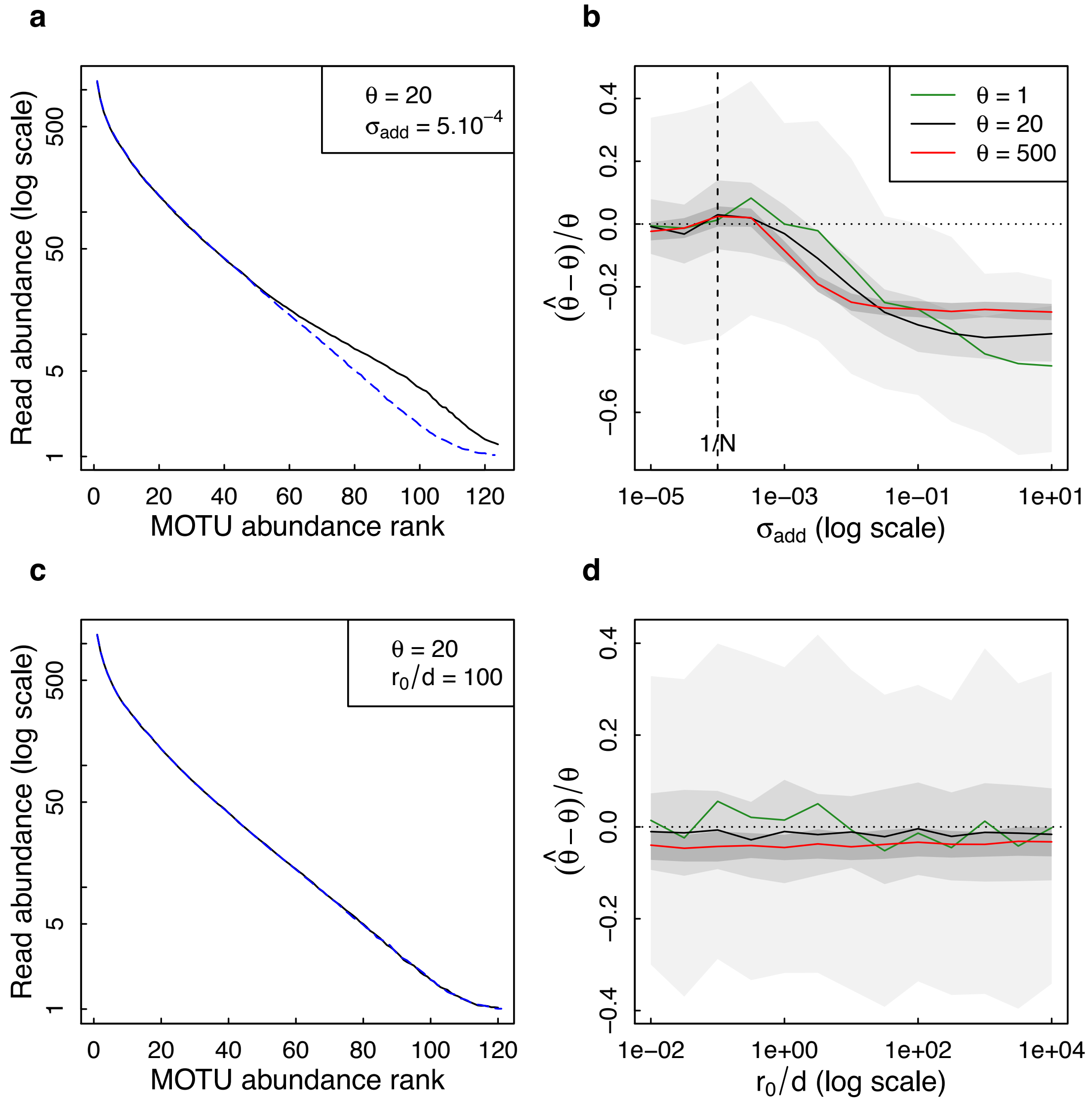

Figure S1: Effect of additive noise and metabolic rate on neutral parameter inference. **Left panels**: mean MOTU rank abundance distributions over 100 realizations for $\theta = 20$ in a 10^4-read sample, without (dashed blue line) and with (black line) simulated noise: (**a**) additive Gaussian noise of standard deviation $\sigma_{add} = 5.10^{-4}$ (5 times the relative abundance $1/N = 10^{-4}$ of the least abundant MOTUs), and (**c**) size structure among individuals and non-linear scaling of DNA release with body mass, for a body size ratio $\frac{g}{dn_0} = 1{,}000$ and a ratio $\frac{r_0}{d} = 100$ between metabolic rate and death rate. **Right panels**: mean and standard deviation over 100 realizations of the relative bias on the θ estimate in a 10^4-read sample, for $\theta = 1$ (green), $\theta = 20$ (black) and $\theta = 500$ (red), as a function of (**b**) the additive noise intensity σ_{add}, and (**d**) the ratio $\frac{r_0}{d}$.

Supplementary Figure 2

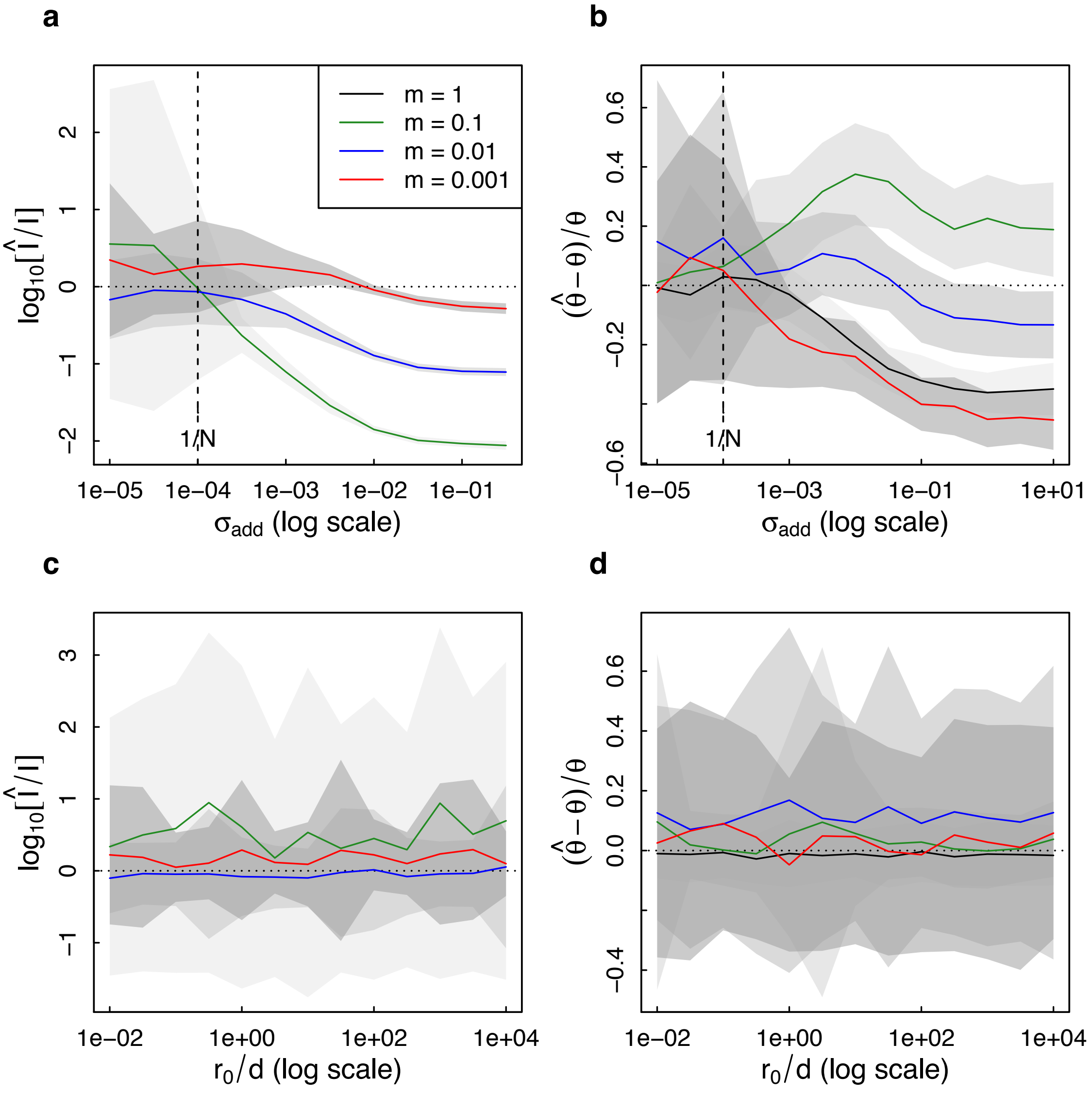

Figure S2: Effect of additive noise and metabolic rate on neutral parameter inference in the presence of dispersal limitation. We simulated a 10^4-read sample and computed the mean and standard deviation over 100 realizations of $(\hat{\theta} - \theta)/\theta$ and $\log_{10}(\hat{I}/I)$. Results are plotted for $\theta = 20$ and for $m = 1$ (black), $m = 0.1$ (green), $m = 0.01$ (blue) and $m = 0.001$ (red). **Panels a-b:** variation with the noise intensity σ_{add} of an additive Gaussian noise on relative abundances ($1/N = 10^{-4}$ is the relative abundance of the least abundant MOTUs). **Panels c-d:** variation with the ratio $\frac{r_0}{d}$ between metabolic rate and death rate.

Supplementary Methods: Quantifying noise using a benchmark dataset

To build our benchmark dataset, we mixed the genomic DNA extracted from 16 Alpine plant species in known quantities (Table S1), and we amplified and sequenced the chloroplast *trn*L P6-loop barcode (primer g-h) [1]. Amplification and sequencing were replicated eight times. The DNA concentrations of the different species in the mixture scaled logarithmically, with a doubling in genomic DNA concentration from one species to the next more abundant. The 16 species thus spanned a large range of DNA concentration (1.10^{-5} ng/µL to 1 ng/µL), representative of the DNA abundances found in environmental samples.

The PCR mixtures comprised 2 ng DNA template, 10 µl of AmpliTaq Gold® Master Mix (Life Technologies, Carlsbad, CA, USA), 0.25 µM of each primer, 3.2 µg of BSA (Roche Diagnostic, Basel, Switzerland) for a final reaction volume of 20 µl. Thermocycling conditions consisted of an initial denaturation step (95°C, 10 min) followed by 35 cycles of denaturation at 95°C (30 s), primer annealing at 50°C (30 s) and elongation at 72°C (1 min), and by a final extension step (72°C, 7 min). Amplicons were then purified (MinElute™ PCR purification kit, Qiagen), pooled, loaded on a HiSeq Illumina lane and sequenced using the paired-end technology. The read coverage was about 10^5 Illumina sequence reads for each of the eight replicates.

The sequencing data were first curated following classical procedures using the OBITools package[2], consisting in paired-end read assembly, read assignation to their respective samples and dereplication. Sequences of length shorter than 10 nucleotides or containing ambiguous nucleotides were excluded. The sequences were then processed using the Infomap clustering algorithm[3], to minimize the number of artifactual MOTUs by clustering sequences together based on their similarity. The dataset is considered as a network of sequences connected by links weighted according to sequence similarity. We used weights decreasing exponentially with the number of nucleotide differences between sequences and we discarded the links for more than 5 nucleotide differences. All replicates were lumped for this clustering analysis. In parallel, all sequences were assigned to a taxon using the barcodes of the 16 species as a reference database (Table S1).

The clustering algorithm yielded 48 clusters (i.e. MOTUs), 24 of which were found only in some of the replicates (Fig. S3a). Each input species was represented as the most abundant sequence of a MOTU found in all 8 realizations. Taking only into account the MOTUs shared across replicates, the proportion of artifactual MOTUs in the curated dataset is 33% (Fig. S3b). Using the taxonomic assignation of all sequences to the most similar of the 16 species, we found that each artifactual MOTU originates from a single species and is at least 50 times less abundant than the species that generated it (Fig. S3a). Therefore, artifactual MOTUs have little impact on the abundance of the true MOTUs in the dataset. Moreover, the number of artifactual MOTUs generated by a species is proportional to the latter's read abundance r (Fig. S3c), and the log-abundance

of these artifactual MOTUs is uniformly distributed between 0 and $\log(r/50)$. Our modeling choice for simulating artifactual MOTUs with realistic abundances built on these empirical observations.

The amplification factor, i.e. the ratio between the read abundance and the initial DNA concentration, was found to be approximately constant over the range of DNA concentrations spanned in the dataset (Fig. S3d). However, it varied across species and replicates. This results in a multiplicative noise on relative abundances that is approximately lognormally distributed, with logarithm standard deviation $\sigma_{log} = 1.2$ (Fig. S3e). Seventy-three percent of the variance of the logarithm is explained by differences among species (likely related to the variability in barcode copy number and in efficiency of PCR amplification) while the remaining variance corresponds to the variability among realizations (Fig. S3d).

References:

1. Taberlet, P. *et al*. Power and limitations of the chloroplast *trn*L (UAA) intron for plant DNA barcoding. *Nucleic Acids Res*. **35,** e14 (2007).

2. Boyer, F. *et al.* OBITOOLS: a UNIX-inspired software package for DNA metabarcoding. *Mol. Ecol. Res.* **16,** 176-182 (2016).

3. Rosvall, M., Axelsson, D. & Bergstrom, C. T. The map equation. *Eur. Phys. J.* **178,** 13-23 (2009).

Species	Dilution factor	Sequence	Sequence length (nt)	Sequence GC content (%)
Taxus baccata	1.000000	atccgtattataggaacaataattttattttctagaaaagg	41	24.39
Salvia pratensis	0.500000	atcctgttttctcaaaacaaaggttcaaaaaacgaaaaaaaaaag	45	26.67
Populus tremula	0.250000	atcctatttttcgaaaacaaacaaaaaaacaaacaaaggttcataaagacagaataagaatacaaaag	68	25.00
Rumex acetosa	0.125000	ctcctcctttccaaaaggaagaataaaaaag	31	35.48
Carpinus betulus	0.062500	atcctgttttcccaaaacaaataaaacaaatttaagggttcataaagcgagaataaaaaag	61	27.87
Fraxinus excelsior	0.031250	atcctgttttcccaaaacaaaggttcagaaagaaaaaag	39	33.33
Picea abies	0.015625	atccggttcatggagacaatagtttcttcttttattctcctaagataggaaggg	54	38.89
Lonicera xylosteum	0.007813	atccagttttccgaaaacaagggtttagaaagcaaaaatcaaaaag	46	32.61
Abies alba	0.003906	atccggttcatagagaaaagggtttctctccttctcctaaggaaagg	47	44.68
Acer campestre	0.001953	atcctgttttacgagaataaaacaaagcaaacaagggttcagaaagcgagaaaggg	56	39.29
Briza media	0.000977	atccgtgttttgagaaaacaagggggttctcgaactagaatacaaaggaaaag	53	39.62
Rosa canina	0.000488	atcccgttttatgaaaacaaacaaggtttcagaaagcgagaataaataaag	51	31.37
Capsella bursa-pastoris	0.000244	atcctggtttacgcgaacacaccggagtttacaaagcgagaaaaaagg	48	45.83
Geranium robertianum	0.000122	atcctttttacgaaaataaagaggggctcacaaagcgagaatagaaaaaaag	53	33.96
Rhododendron ferrugineum	0.000061	atcctttttcgcaaacaaacaaagattccgaaagctaaaaaaaag	46	30.43
Lotus corniculatus	0.000031	atcctgctttacgaaaacaagggaaagttcagttaagaaagcgacgagaaaaatg	55	38.18

Table S1: List and characteristics of the 16 plant species included in the benchmark dataset.

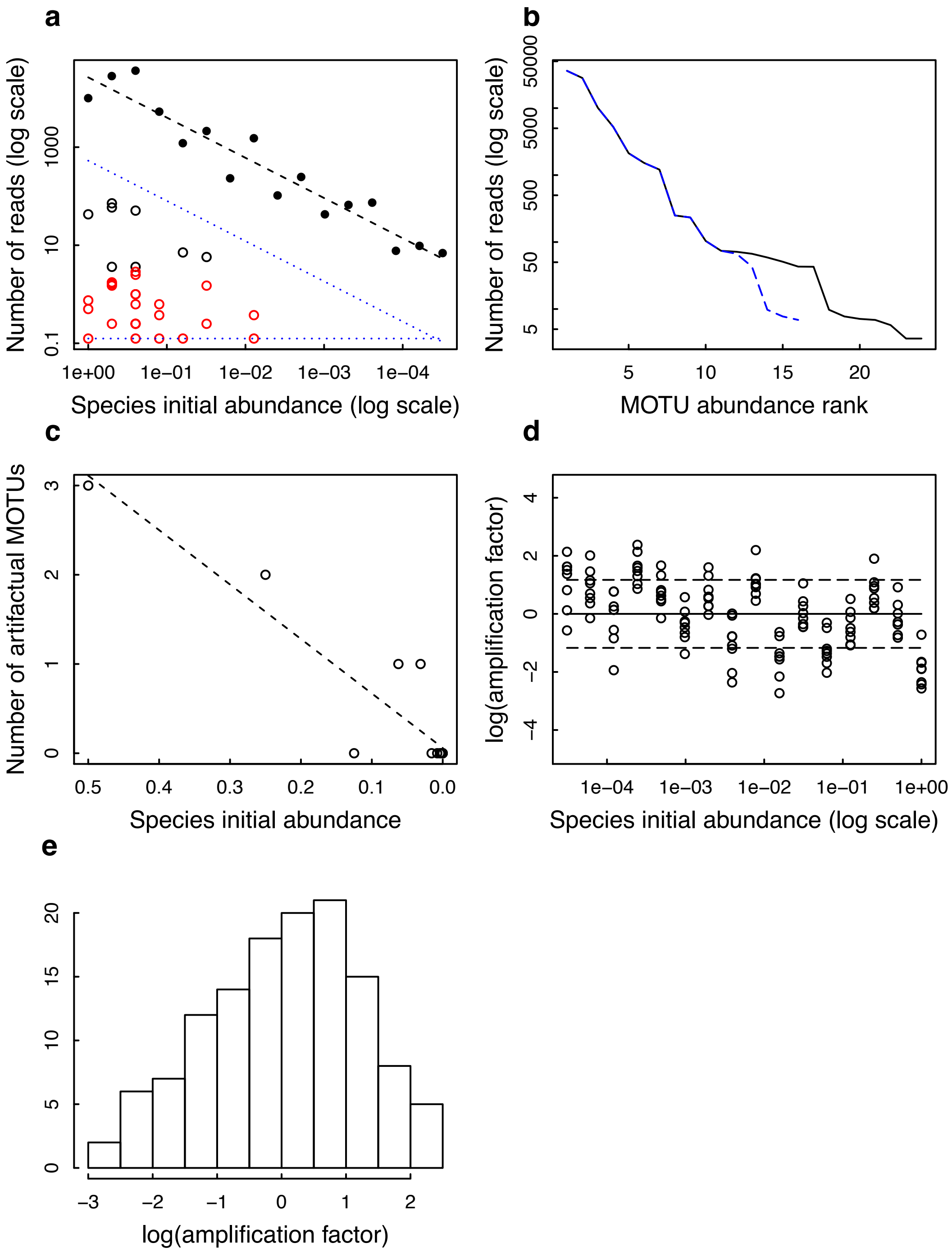

Figure S3: Empirical results for the benchmark dataset obtained by mixing the DNA of 16 plant species, then amplifying by PCR and sequencing on an Illumina platform the chloroplast trnL P6-loop barcode, with eight replicates. **Panel a**: Read abundance of the 16 species (•) and of the artifactual MOTUs (∘, ∘), averaged over the replicates, as a function of the species initial abundance. Some artifactual MOTUs were found in every realization (∘), but others were not (∘). The blue dotted lines delineate the abundance domain chosen to model the abundances of artifactual MOTUs. **Panel b**: Number of reads per MOTU as a function of the MOTU's abundance rank, including and excluding artifactual MOTUs (black and dashed blue, respectively). **Panel c**: Linear relationship between the number of artifactual MOTUs and the relative abundance of the species that

generated them (the most abundant species is excluded, as well as the MOTUs found in only some of the replicates). **Panel d**: Logarithm of the amplification factor, i.e. the ratio between the read abundance and the initial DNA concentration, as a function of the initial DNA concentration of the species (dotted lines: standard deviation $\sigma_{log} = 1.2$ over all species and all replicates). **Panel e**: Probability density of the logarithm of the amplification factor over the 16 species and the 8 realizations, approximately normally distributed.

Supplementary Note 1: Hubbell's neutral model

Hubbell's neutral model of biodiversity describes a large pool of J_M individuals undergoing random death, birth and speciation events in the following way: at each time step, one individual at random dies, and is replaced by a new individual. This new individual belongs to a taxon not previously found in the community with probability ν, or to one of the already existing taxa with probability $1-\nu$. In the latter case, each taxon has a probability to be picked proportionally to its abundance in the community[1]. In the absence of dispersal limitation, the multivariate steady-state distribution of taxa abundances is called the Ewens distribution and is characterized by the single parameter $\theta = \frac{\nu}{1-\nu}(J_M - 1)$ [2,3]. Any sample consisting of $J < J_M$ individuals drawn at random from the community follows also the Ewens distribution of parameter θ.

A dispersal-limited version of this model is defined as follows[1,3]. New taxa disperse into a single local community by immigration from a regional pool, which follows the model without dispersal limitation described above. When an individual dies, it is replaced by an immigrating individual with probability m, and by the offspring of a local individual with probability $1-m$. Two immigrants may belong to the same taxon. The multivariate steady-state distribution of taxa abundances in the dispersal-limited local community depends on two parameters: the dispersal parameter $I = \frac{m}{1-m}(J-1)$, where J is the number of individuals in the local community, and the diversity parameter θ of the regional pool[4]. Any sample drawn at random from the local community also follows the Etienne distribution of parameters θ and I [3].

References:

1. Hubbell, S. P. *The Unified Neutral Theory of Biodiversity and Biogeography*. (Princeton Univ. Press, 2001).

2. Ewens, W. J. The sampling theory of selectively neutral alleles. *Theor. Popul. Biol.* **3,** 87-112 (1972).

3. Etienne, R. S. & Alonso, D. A dispersal-limited sampling theory for species and alleles. *Eco. Let.* **8,** 1147-1156 (2005).

4. Etienne, R. S. A new sampling formula for neutral biodiversity. *Eco. Let.* **8,** 253-260 (2005).

Supplementary Note 2: Modeling size differences

1. *Modeling size differences using the von Foerster equation*

The von Foerster equation[1,2] describes a population where individuals grow in number of cells (or mass) n with a growth rate $g(n)$, and where they die with a death rate $d(n)$. The evolution of the number $j(n,t)\mathrm{d}n$ of individuals with a number of cells between n and n+dn at time t is given by:

$$\frac{\partial j(n,t)}{\partial t} = -\frac{\partial\big(g(n)j(n,t)\big)}{\partial n} - d(n)j(n,t)$$

When $g(n)$ and $d(n)$ are independent of n, the stationary (i.e., time-independent) solution of the von Forster equation is:

$$j(n) = J\frac{d}{g}e^{-\frac{d}{g}(n-n_0)}$$

, where J is the constant population size, given by:

$$J = \int_{n_0}^{\infty} j(n)\, dn$$

Therefore, a randomly chosen individual in the population has a number n of cells with probability density:

$$p_{ind}(n) = \frac{j(n)}{J} = \frac{d}{g}e^{-\frac{d}{g}(n-n_0)}$$

We used this probability density to draw a number of cells between n_0 and infinity for each individual of the neutral sample. The mean $< n >$ and the coefficient of variation $\sigma_n/< n >$ of the number of cells of a randomly chosen individual are given by:

$$< n > = \frac{g}{d} + n_0$$

$$\frac{\sigma_n}{< n >} = \frac{1}{\frac{d}{g}n_0 + 1}$$

2. *Comparison with O'Dwyer et al. (2009)'s size-structured neutral model*

O'Dwyer *et al.* (2009)[2] transformed the deterministic von Foerster equation into a probabilistic equation, and integrated it into the master equation of Volkov *et al.* (2003)[3], which describes a neutral dynamics without dispersal limitation in a probabilistic way. The resulting size-structured neutral model predicts that, in steady state, if the growth rate $g(n)$, the birth rate $b(n)$ and the death rate $d(n)$ are independent of n, and if $\frac{g}{d} \gg n_0$ (i.e., individuals grow much larger than their size at birth), a randomly chosen species will have a total number of cells (or a total biomass) n with probability density:

$$p_{sp}(n) = \frac{\nu}{bn}\left(e^{-\frac{d-b}{g}n} - e^{-\frac{d}{g}n}\right)$$

where ν is the speciation rate of the neutral model ($\nu/b \ll 1$). Adding size structure does not modify the probability for a randomly chosen species to have J individuals:

$$P_{sp}(J) = \frac{\nu}{bJ}\left(\frac{b}{d}\right)^J$$

While the model of O'Dwyer *et al.* (2009)[2] explicitly accounts for the coupling between the demographic dynamics and the growth of individuals, we generated a neutral sample of individuals and then assigned an independent number of cells to each individual. Therefore, under our assumptions, the numbers of cells of the different individuals are described by independent and identically distributed exponential random variables N_i, and for $\frac{g}{d} \gg n_0$, the total number of cells of a species with J individuals follows an Erlang distribution:

$$\sum_{i=1}^{J} N_i \sim Erlang(\frac{g}{d}, J)$$

with probability density:

$$p_{sp}(n|J) = p_{Erlang(\frac{g}{d},J)}(n) = \frac{1}{(J-1)!}\left(\frac{d}{g}\right)^J n^{J-1} e^{-\frac{d}{g}n}$$

The probability density for a species of having a total number of cells *n* is then given by:

$$p_{sp}(n) = \sum_{J=1}^{\infty} P_{sp}(J) p_{sp}(n|J)$$

Combining the expressions of $P_{sp}(J)$ and $p_{sp}(n|J)$ above, we obtain the same expression for $p_{sp}(n)$ as predicted by the size-structured model of O'Dwyer *et al.* (2009)[2]. Therefore, in the simple case where $g(n)$, $b(n)$ and $d(n)$ are independent of the number of cells *n*, explicitly accounting for the coupling between demographic dynamics and individual growth is equivalent to assuming as we did that all individuals have independent and identically distributed numbers of cells.

The modelling approach of Volkov *et al.* (2003)[3] and O'Dwyer *et al.* (2009)[2] differs from that of Ewens (1972)[4] and Etienne (2005)[5]. The former consists in describing the population dynamics of a single species with a fluctuating number of individuals, independently of the remaining of the community, and then considering that the results hold for every species in the community ("mean-field" approach). In contrast, the Ewens and Etienne distributions are obtained by explicitly considering a community with a constant number of individuals and a fluctuating number of species through time. However, the two approaches yield identical stationary distributions provided that the number of species is large enough[6].

References:

1. Von Foerster, H. Some remarks on changing populations in *Kinetics of Cellular Proliferation* (ed. Stohlman, F.), 382–399 (Grune and Stratton, 1959).

2. O'Dwyer, J. P. Lake, J. K., Ostling, A., Savage, V. M. & Green, J. L. An integrative framework for stochastic, size-structured community assembly. *PNAS*. **106,** 6170-6175 (2009).

3. Volkov, I., Banavar, J. R., Hubbell, S. P. & Maritan, A. Neutral theory and relative species abundance in ecology. *Nature.* **424,** 1035-1037 (2003).

4. Ewens, W. J. The sampling theory of selectively neutral alleles. *Theor. Popul. Biol.* **3,** 87-112 (1972).

5. Etienne, R. S. A new sampling formula for neutral biodiversity. *Eco. Let.* **8,** 253-260 (2005).

6. Etienne, R. S., Alonso, D. & McKane, A. J. The zero-sum assumption in neutral biodiversity theory. *J. of Theor. Biol*. **248,** 522-536 (2007).

Supplementary Note 3: Estimator performance without simulated noise

We explored how the maximum-likelihood neutral estimators behave in the absence of simulated noise over the range of tested parameter values (θ in [1, 500] and m in [0.001, 1]). We found that while the Ewens estimator is very little biased (Fig. S4a-b), the dispersal-limited estimator can be strongly biased depending on parameter values and sample size (Fig. S4c-f). The dispersal-limited estimator underestimates θ and overestimates I when the immigration rate into the local community is too small, and overestimates θ and underestimates I when the immigration rate is too large. In the case of our 10^4-read sample, values of I around $I = 10^3$ (i.e. $m = 0.01$ in the 10^5-individual sample) allow for the least biased estimation of (θ, I). Biases are strongest for $\theta > 100$.

For both estimators standard deviation and bias decrease with sample size, but a much larger sample size is required to obtain accurate estimates in the dispersal-limited case than in the absence of dispersal limitation. While sample sizes of ca. $N = 100$ are sufficient for the Ewens estimator, sample sizes of $N = 10^4$ are still not sufficient for some parameter values in the dispersal-limited case. Larger θ values and smaller I values require larger sample sizes. Estimating the neutral parameters simultaneously from several read samples reduces these biases[1].

Reference:

1. Etienne, R. S. A neutral sampling formula for multiple samples and an 'exact' test of neutrality. *Eco. Let.* **10,** 608-618 (2007).

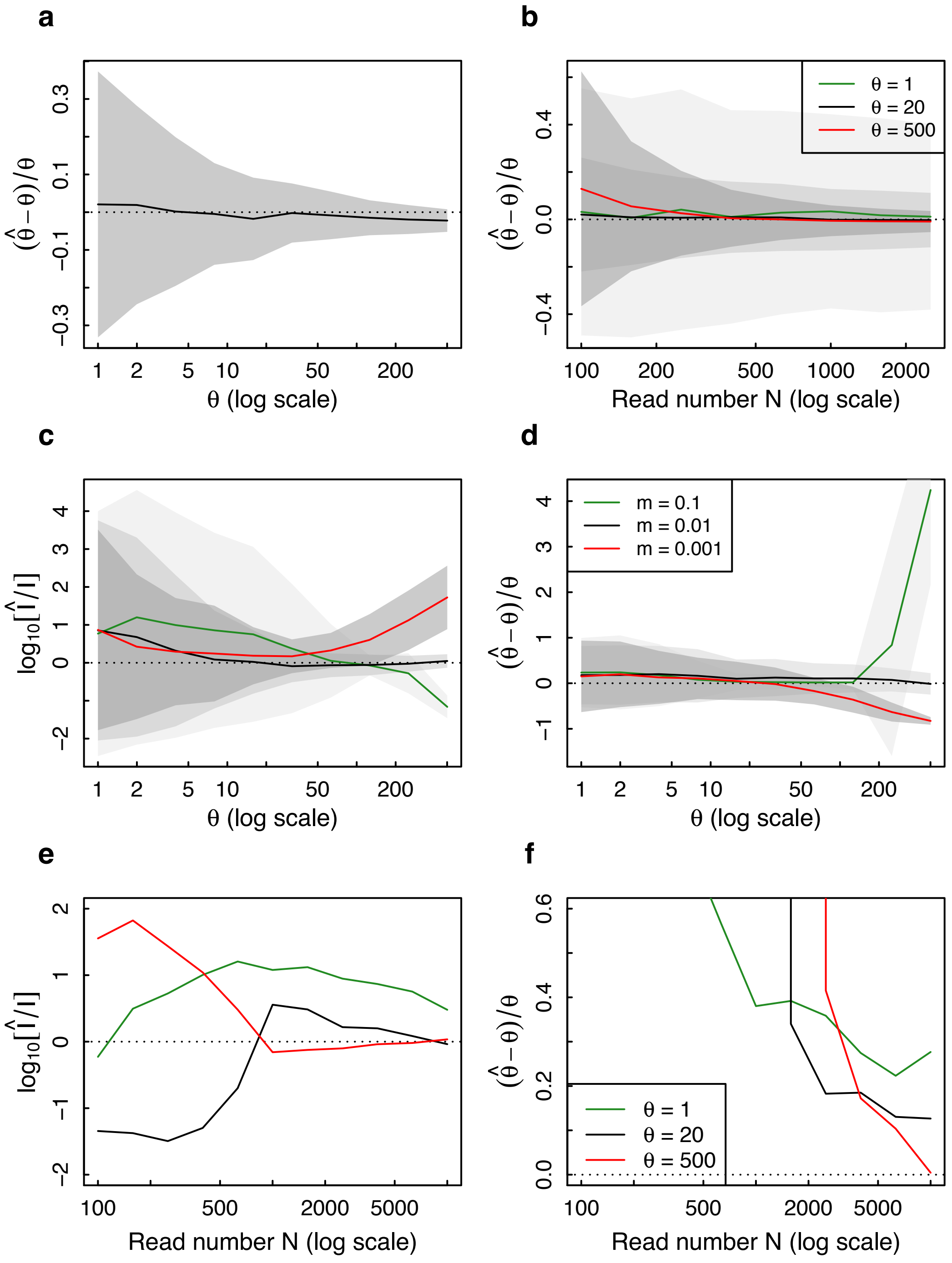

Figure S4: Neutral parameter inference without simulated noise, for different parameter values. The mean and standard deviation of the relative biases on parameter estimates are plotted over 500 realizations. **Panels a-b:** θ inference without dispersal limitation, as a function of (a) the input θ value and (b) the read number N, for θ equal to 1, 20, and 500. **Panels c-d:** θ and $\log_{10}(I)$ inference as a function of the input θ value, for m equal to 0.1, 0.01, and 0.001. **Panels e-f:** θ and $\log_{10}(I)$ inference as a function of the read number N, for $m = 0.01$ and for θ equal to 1, 20, and 500.

Supplementary Note 4: Neutral parameter inference with the number of individuals unknown

Because exact maximum-likelihood inference of the neutral parameters relies on sampling formulas that are invariant under subsampling, it is possible to use the sequence reads as effective individuals as long as we can consider the reads as a subsample from the initial individuals. Therefore, there should be less reads than individuals. A further complication is that the sequence reads are sampled with replacement from the initial individuals in our simulations (i.e. they are a multinomial sample from the relative abundances) instead of without replacement as required for the invariance property to hold. Hence there should be in fact several times less reads than individuals, because sampling with and without replacement are equivalent only in this case.

To illustrate this assumption, we explored how the Ewens maximum-likelihood estimator behaves in the absence of simulated noise depending on the initial number of individuals J, for $N = 10^2, N = 10^3, N = 10^4$ and $N = 10^5$, and for $\theta = 20$. As expected, the Ewens estimator yields an unbiased θ estimate as long as the initial number of individuals is ca. one order of magnitude larger than the number of reads (Fig. S5a-d). We then simulated a number of reads larger than the initial number of individuals ($N = 10^5$ reads for $J = 10^4$ individuals, and $N = 10^4$ reads for $J = 10^3$ individuals), and took smaller subsamples of reads from the original read sample until reaching a stable θ maximum-likelihood estimate. As expected, the θ estimate becomes stable under subsampling for samples at least one order of magnitude smaller than the initial number J of individuals. Using this method, we achieved an unbiased estimation of θ in spite of the small initial number of individuals (Fig. S5e-f). In the dispersal-limited case, we expect the maximum likelihood estimator based on the Etienne sampling formula to behave similarly.

We also compared estimating θ using the Ewens estimator and estimating θ by linear regression on the ranked log-abundance. We found that both methods perform similarly when the number of reads is one order of magnitude larger than the initial number of individuals, and that when this condition is not met, linear regression still provides an unbiased θ estimate (Fig. S5a-d). However, unlike maximum likelihood inference, linear regression on the ranked log-abundance is not reliable when either the number of reads or the initial number of individuals is too low (lower than ca. 500 for $\theta = 20$; Fig. S5a-d), or when there is too little taxonomic diversity in the sample. Moreover, the θ estimate depends on the arbitrary delimitation of the linear domain of the curve.

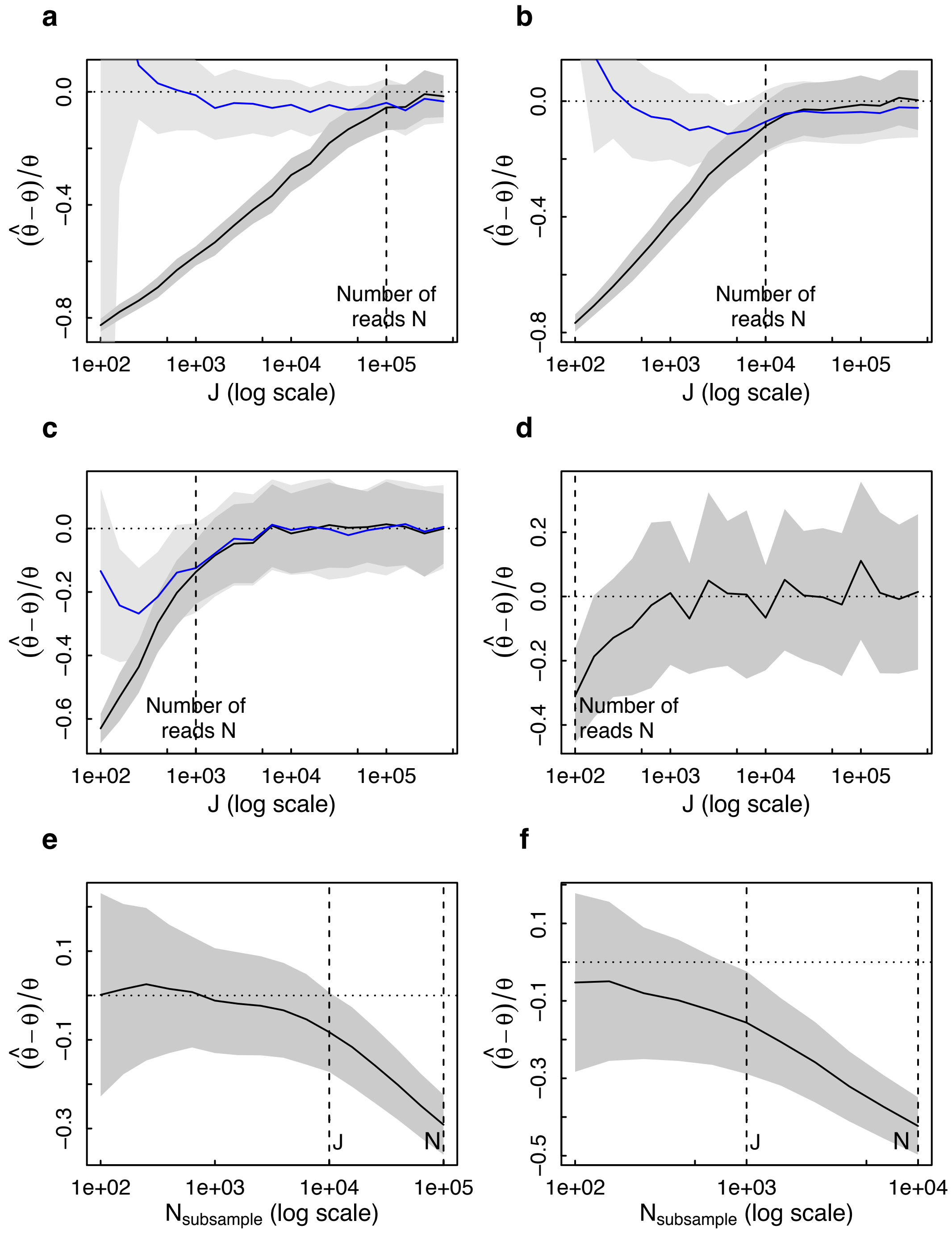

Figure S5: θ inference without dispersal limitation and without simulated noise for $\theta = 20$. The mean and standard deviation of the relative bias on the θ estimate are plotted over 100 realizations. **Panels a-d:** θ inference by maximum likelihood (black) and by linear regression on the ranked log-abundance (blue), as a function of the initial number of individuals J, (a) for $N = 10^5$ reads, (b) $N = 10^4$ reads, (c) $N = 10^3$ reads and (d) $N = 10^2$ reads (linear regression too inaccurate to be plotted for $N = 10^2$)**. Panels e-f:** Maximum-likelihood θ estimate as a function of the size $N_{subsample}$ of the read subsample used for estimation, starting from an original sample of (e) $N = 10^5$ reads or (f) $N = 10^4$ reads. An unbiased θ estimate is obtained when $N_{subsample}$ is at least one

order of magnitude smaller than the initial number of individuals (e) $J = 10^4$ or (f) $J = 10^3$.
